# Spermidine attenuates chondrocyte inflammation and cellular pyroptosis through the AhR/NF-κB axis and the NLRP3/caspase-1/GSDMD pathway

**DOI:** 10.3389/fimmu.2024.1462777

**Published:** 2024-10-02

**Authors:** Xiaocheng Guo, Xinyuan Feng, Yue Yang, He Zhang, Lunhao Bai

**Affiliations:** Department of Orthopedics, Shengjing Hospital of China Medical University, Shenyang, China

**Keywords:** spermidine, osteoarthritis, aromatic hydrocarbon receptor, inflammation, pyroptosis

## Abstract

**Introduction:**

Osteoarthritis (OA) is a prevalent chronic degenerative disease, marked by a complex interplay of mechanical stress, inflammation, and metabolic imbalances. Recent studies have highlighted the potential of spermidine (SPD), a naturally occurring polyamine known for its anti-inflammatory and antioxidant properties, as a promising therapeutic agent for OA. This study delves into the therapeutic efficacy and mechanistic pathways of SPD in mitigating OA symptoms.

**Methods:**

Forty Sprague-Dawley rats were randomly assigned to four groups, including the CG (sham operation), model (anterior cruciate ligament transection [ACLT], and treatment (ACLT + two different doses of SPD) groups. *In vivo*, correlations between OA severity and different interventions were assessed by ELISA, X-rays, CT imaging, histological staining, and immunohistochemistry. *In vitro*, IL-1β was used to trigger chondrocyte inflammation, and SPD’s cytotoxicity was assessed in primary rat chondrocytes. Next, inflammatory markers, extracellular matrix (ECM) proteins, and pathway marker proteins were detected in chondrocytes administered IL-1β alone, SPD, or aryl hydrocarbon receptor (AhR) silencing, by qRT-PCR, Griess reaction, ELISA, Western blot, and immunofluorescence. Morphological alterations and pyroptosis in chondrocytes were examined by transmission electron microscopy (TEM) and flow cytometry.

**Results:**

Our research reveals that SPD exerts significant anti-inflammatory and antipyroptotic effects on IL-1β-treated chondrocytes and in anterior cruciate ligament transection (ACLT) rat models of OA, primarily through interaction with the Aryl hydrocarbon receptor (AhR). Specifically, SPD’s binding to AhR plays a crucial role in modulating the inflammatory response and cellular pyroptosis by inhibiting both the AhR/NF-κB and NLRP3/caspase-1/GSDMD signaling pathways. Furthermore, the knockdown of AhR was found to negate the beneficial effects of SPD, underscoring the centrality of the AhR pathway in SPD’s action mechanism. Additionally, SPD was observed to promote the preservation of cartilage integrity and suppress ECM degradation, further supporting its potential as an effective intervention for OA.

**Discussion:**

Collectively, our findings propose SPD as a novel therapeutic approach for OA treatment, targeting the AhR pathway to counteract the disease’s progression and highlighting the need for further clinical evaluation to fully establish its therapeutic utility.

## 1 Introduction

Osteoarthritis (OA) causes pain and disability and imposes an economic burden on patients and the society. The incidence of OA is on the rise due to increasing numbers of elderly and obese individuals worldwide (1). In OA, the entire joint is affected by alterations in periarticular muscles, synovium, articular cartilage, ligaments, subchondral bone, and joint capsule, and such effects are not limited to wear and tear and degenerative diseases (2, 3). Current research identifies low-grade inflammation, which leads to disrupted anabolic and catabolic processes, as a key contributor to OA. Studies have shown that levels of inflammatory mediators, such as IL-6, IL-1β, COX-2, and PGE2, undergo significant changes throughout the progression of OA (4); therefore, attenuating IL-1β-induced inflammation in chondrocytes may offer a potential treatment strategy for osteoarthritis.

Spermidine (SPD), a commonly expressed polyamine, represents a potential drug for OA and a natural autophagy agonist. The anti-aging, anti-inflammatory, cardiovascular, and neuromodulatory effects of SPD are well documented (5, 6). In recent years, with advances in metabolomics, decreased ratios of spermine to spermidine have been reported in serum samples from OA patients (7), which may be associated with OA severity; SPD is an essential molecule in the cycle that regulates polyamine metabolism. SPD enhances cardiomyocyte autophagy and improves cardiac function (8), attenuates pulmonary fibrosis by downregulating pro-inflammatory cytokines in alveolar epithelial cells (9), modulates neuroglial cell-mediated neuroinflammatory responses (10), exerts a beneficial effect on verbal memory and inflammation (11), and induces anti-inflammatory macrophages and regulates intestinal dysbiosis to alleviate colitis (12). Consequently, while SPD is known for its anti-inflammatory properties across various tissues, its precise impact on inflammatory chondrocytes and the pathogenesis of OA has yet to be fully understood. This study seeks to elucidate the anti-inflammatory potential of SPD specifically within the context of OA.

The aryl hydrocarbon receptor (AhR), a member of the basic helix-loop-helix (bHLH)-PAS family of transcription factors, acts as a crucial environmental sensor. These receptors play pivotal roles in responding to endogenous and exogenous signals, influencing a wide range of biological processes (13). AhR forms cytoplasmic complexes with HSP90, XAP2, and p23 in the absence of a ligand. Following ligand binding, the AhR molecule dimerizes with AhR nuclear translocator (ARNT) (14). Although previously considered a mediator of environmental pollutants, the understanding of AhR function in immune cells and the discovery of “natural” AhR ligands have led to the notion that AhR activation is beneficial. AhR signaling induction involves endogenous and exogenous ligands, which may be found in the environment or synthesized metabolically (15). In a study on the aryl hydrocarbon receptor, it was shown that increased levels of polyamines can upregulate AhR receptor binding to DNA in a concentration-dependent manner (16). Interestingly, the work of chen et al. found that SPD could inhibit the progression of osteoarthritis in synoviocytes by activating the deubiquitination of RIP1 (Receptor-interacting protein 1) (17). However, the specific mechanism of SPD on osteoarthritis is still unclear. We hypothesized that SPD protects chondrocytes and alleviates OA via AhR.

Inflammation plays an essential role in OA development. Evidence suggests that in the early stages of OA, although articular cartilage has not yet undergone significant changes, subchondral osteosclerosis causes an imbalance in the subchondral bone (18), which induces synovial tissues to release pro-inflammatory factors, leading to extracellular matrix (ECM) degradation of articular cartilage (19). Eventually, abnormal acceleration of articular chondrocyte catabolism drives ECM degradation over synthesis, becoming the major feature of OA cartilage (20). Nuclear factor-κB (NF-κB) represents a class of transcription factors known to be induced by a variety of pro-inflammatory cytokines (21). It is currently admitted NF-κB signaling is widely considered a target for OA therapy (22). Proinflammatory factors can trigger cellular pyroptosis, which is associated with chondrocytes’ NF-κB activity and osteoarthritis (23). Pyroptosis is a pro-inflammatory programmed cell death activated by nod-like receptor protein 3 (NLRP3)/caspase-1, accompanied by the cleavage of gasdermin D (GSDMD), which oligomerizes its N-terminal structural domain and generates a pore in the cell membrane, inducing cell death (24). These observations underpin the hypothesis that SPD could modulate inflammatory responses and reduce pyroptosis in OA through impacting the NLRP3/caspase-1/GSDMD pathway. Such a mechanism suggests a potential therapeutic role for SPD in mitigating the inflammatory and pyroptotic processes associated with OA, providing new perspectives on OA treatment.

## 2 Materials and methods

### 2.1 Network pharmacology and target acquisition

First, we retrieved osteoarthritis-related target information from Genecards (https://www.genecards.org/) and Disgenet (https://www.disgenet.org/). Then, the structural information of spermidine (Compound CID: 1102) was obtained from PubChem (https://pubchem.ncbi.nlm.nih.gov/), and target prediction analysis was performed with the GalaxySagittarius software (25). Based on these analyses, the top 300 targets with highest target prediction scores were considered potential targets for the effects of SPD and cross-referenced with the target data of osteoarthritis to identify the key targets of SPD that might affect the disease.

To further examine these potential targets, KEGG and GO enrichment analyses were performed, and a protein-protein interactions (PPI) network was generated with the STRING database (https://string-db.org/), aiming to determine key targets closely related to the pathological process of osteoarthritis and to provide a direction for the selection of major targets in subsequent experiments.

To study the binding of small molecules and target proteins, molecular docking data were imported into the Maestro 13.5 software, in which the Interactions Toggle and 2D Sketcher modules were employed to analyze in detail key interaction points between ligands and receptors such as hydrogen bonding and hydrophobic interactions.

Subsequently, an 80-ns molecular dynamics (MD) simulation of the interactions between the key targets and SPD was carried out with the Gromacs software package (26) using the Charmm36 force field and the TIP3P water model. Charge neutralization was achieved by adding appropriate amounts of Na+ and Cl- to the simulated system, and energy minimization was performed to eliminate undesirable interactions. The most rapid descent method was employed for energy minimization, setting the maximum number of steps to 5000 and the force tolerance to 1000 kJ/mol-nm. After completing energy minimization, we performed equilibrium simulations of the system with constant volume/constant temperature (NVT) and constant pressure/constant temperature (NPT). Finally, the results of the molecular dynamics simulations were examined with the analysis tool in Gromacs to assess root-mean-square-difference (RMSD) parameters (27) for the SPD molecule concerning the target protein.

### 2.2 Animal experiments

Forty male specific-pathogen-free SD rats (4 weeks old, 220 ± 5 g) were acquired from HFK Biotechnology (China). This study had approval from the Ethics Committee of China Medical University (Approval No. 2021PS130K (X1). The animals were randomly assigned to four groups, including the sham, ACLT, low-SPD (2.5 mg/kg), and high-SPD (5.0 mg/kg), with ten rats in each group. The sample size for each group (n=10) was determined based on a power analysis, ensuring a statistical power of at least 80% to detect significant differences between groups, while maintaining an alpha level of 0.05 (28). This estimation also took into account the variability observed in preliminary experiments and similar published studies (29). Animals were first acclimatized for one week in a tightly controlled feeding environment (12-h photoperiod, 22 ± 2°C and 70% humidity) with ad libitum feed and drinking water. Assays involving animals followed the NIH Guide for the Care and Use of Laboratory Animals, with additional measures to control interindividual variability, such as randomized group assignment and standardized treatment protocols.

### 2.3 OA model establishment and SPD interventions

After anesthesia with pentobarbital sodium (1.0%, 2.5 mL/100 g), anterior cruciate ligament transection (ACLT) was performed in the ACLT and SPD groups to establish an OA rat model, while the sham group was only incised into the medial knee capsule. The low- and high-SPD groups underwent intraperitoneal injection with 2.5 mg/kg and 5.0 mg/kg SPD (S0266, Sigma-Merck, Germany), respectively, once weekly, while the Sham and ACLT groups were intraperitoneally administered 100 μL of sterile saline. Pharmacological interventions were performed one week after modeling for 6 weeks, and body weights were recorded before each procedure. Rats were sacrificed at the end of week 8 by injection of an overdose of pentobarbital sodium, and knee samples were obtained for subsequent assays.

### 2.4 Plain radiography and Micro-CT

Plain radiography of the knee joint was performed on an X-ray machine (MS FX PRO; BRUKER, USA), and 3D images of the knee joint were obtained by SkyScan 1276 Micro-CT (Bruker, Belgium) and assessed with CTAn 1.15.4. At study end, rats underwent anesthesia by intraperitoneal administration of sodium pentobarbital (40 mg/kg) and were then placed supine. Bilateral ankles from treated SD rats were fixed on a tray with adhesive tape. After separating knee samples, soft tissues were removed and a 48-h fixation was performed with 4% paraformaldehyde. Micro-CT was carried out for all knees at 70 kV, 200 μA, and 0.5 mm resolution. Based on the Osteoarthritis Imaging Scoring System, OA severity was evaluated by imaging changes (e.g., joint space narrowing and articular surface calcification) and 3D image reconstruction (30).

### 2.5 Sample preparation and collection

Blood specimens were freshly collected upon pentobarbital anesthesia before euthanasia. After 2h at ambient, serum separation was performed by centrifugation at 1500 rpm for 15 min. Rat knee joints were dissected, and intra-articular lavage fluid (IALF) samples were obtained from the joint cavity by injecting and recovering 0.3 ml of saline three times with a 1-ml syringe without destroying the joint capsule.

### 2.6 Primary chondrocyte isolation and culture

Cartilage samples were obtained from 4-week-old Sprague-Dawley (SD) rats for primary chondrocyte culture. The rats were euthanized, and cartilage tissues from the femoral head, the femoral condyle and intercondylar fossa of the knee joint were obtained and minced into 1-mm² pieces in PBS. Cartilage pieces were next digested using 7 mg/ml proteinase K (V900887; Sigma, USA) at 1:6 dilution in culture medium for 2 h in a 37°C water bath. Then, 10 mg/ml collagenase D (Cat. No. 11088858001 Roche, USA) was added for digestion at 1:9 dilution in culture medium in a 37°C water bath for 1 h. The whole digestion process was accompanied by a low shaking. Finally, the cell suspension containing chondrocytes was submitted to a 5-min centrifugation at 800 rpm. Isolated chondrocytes were transferred into 25 cm^2^ culture dishes in DMEM/F12 (DMEM/F12; Thermo Fisher Scientific, USA) medium containing 10% fetal bovine serum (FBS, Gibco, 10270-106) and 1% penicillin-streptomycin (Servicebio, G4003) at 5% CO2 and 37°C. Thereafter, the medium was refreshed at three-day intervals; when P0 cells were adherent, they harvested with trypsin (Gibco™, 25300062, USA) after reaching 70-80% confluence. Passaging used a 1:3 ratio. Only 1^st^ or 2^nd^ generation cells were used for subsequent experiments to ensure that the original phenotype was intact.

### 2.7 Pharmaceutical preparations and therapeutic interventions

Totally 1 g of spermidine (SPD) (purity ≥98%) (S0266, Sigma) was dissolved in an appropriate amount of PBS to obtain 1 mM. Previous studies have shown that doses of 1-10 μM of spermidine promote chondrocyte differentiation to hypertrophy and terminal differentiation (31). We initially dissolved 1 g of spermidine in an appropriate amount of PBS to obtain a 1mM stock solution. This concentration was selected to align with preliminary studies that highlighted the efficacy of SPD within the 1-10μM range. However, to thoroughly investigate the dose-dependent effects of SPD on chondrocyte viability and elucidate its potential therapeutic window, we refined the scope of the study by setting the concentration range from 0 to 2μM in cell culture experiments. Before chondrocyte treatment, the cells were first starved for 12 h, to decrease the endogenous polyamine pool, thus highlighting the effects of exogenous SPD pretreatment (32). To induce inflammatory reactions in chondrocytes *in vitro*, the optimal IL-1β concentration of 10 ng/ml was selected for intervention (33). Following a 1-h pretreatment with SPD and co-treatment with IL-1β (10 ng/ml) for 24 h, supernatants and cells were obtained for subsequent assays.

### 2.8 Cell counting Kit-8 assay

The effect of SPD on rat primary chondrocyte viability was quantified with Cell Counting Kit-8 (Beyotime, China, C0038). Chondrocytes were inoculated into 96-well plates at 3×10^3^/well and administered increasing concentrations of SPD (0, 0.01, 0.1, 0.5, 1, and 2μM) at 70% confluence for 12 h and 24 h, respectively. Cells were next pretreated with different SPD concentrations for 1 h based on the results above, followed by IL-1β addition at 10 ng/ml for 24 h. Next, 10 μL of CCK-8 solution was added per well for 2 h at 37°C. Optical density was read at 450 nm using a Gen5 plate reader (BioTek, USA). Experiments were performed in triplicate.

### 2.9 ELISA

Chondrocytes underwent a 24-h incubation with 10 ng/ml IL-1β supplemented with SPD alone or siRNA-AhR. Cell culture supernatants were obtained, and nitric oxide (NO), TNF-α, IL-6, and PGE2 amounts were determined. Additionally, TNF-α, IL-6, and PGE2 in both serum and joint cavity lavage fluid samples from rats were determined. NO concentrations were assessed using the Nitrate/Nitrite Assay Kit (Beyotime, S0024). TNF-α (Multisciences, EK382), IL-6 (Multisciences, EK306), and PGE2 (Multisciences, EK8103) concentrations were obtained by ELISA kits as specified by the manufacturer. A negative control group (chondrocytes with culture medium only, no SPD) was included for each experiment to account for the effect of solvents. A positive control group (chondrocytes treated with IL-1β only), was also included. All treatments were performed in triplicate and results were normalized to those of the negative control group.

### 2.10 siRNA transfection

Three sequences of small interfering RNA targeting AhR (siRNA-AhR) were provided by Hippobio (China). Chondrocytes underwent treatment with 10 ng/ml IL-1β, SPD, or siRNA (50nM) alone. Transfection used Lipofectamine 3000 (Invitrogen, L3000015) according to the manufacturer’s directions. Subsequent assays were performed after 48 h of transfection. siRNA primers are listed in **Table 1**.

**Table 1:** siRNA primers used in this study.

siRNA-AhR	Sequence (5’ → 3’)
rAhr si-1 sense	CCUCCACAGUUGGCUUUGUUUTT
rAhr si-2 sense	GCACGCUUGAUUUACAGAAAUTT
rAhr si-3 sense	CACCCUAACGCUUCUAAUUUATT
Control siRNA	UUCUCCGAACGUGUCACGUdTdT

### 2.11 Plasmid transfections

We used the NF-κB p65 overexpression plasmid to elucidate whether SPD mediates the inhibition of chondrocyte pyroptosis through AhR/NF-κB. When cells reached 60% confluence, primary chondrocytes were transfected with 100ng/ml negative control plasmid (NC) or NF-κB p65 overexpression plasmid (OE) (NM_199267, Syngen Tech Co., Beijing, China) using Lipofectamine 3000 transfection reagent (Invitrogen, L3000015).

### 2.12 Real-time quantitative PCR

Total ribonucleic acid (RNA) was isolated from cell cultures with the RNAiso Plus kit (Vazyme Biotech, China) as directed by the manufacturer. Next, reverse transcription employed the HiScript III Q RT SuperMix for qPCR (+gDNA wiper; Vazyme Biotech). qRT-PCR reactions were prepared with the SYBR Green PCR kit (Vazyme Biotech) and run on a LightCycler 480 (Roche Diagnostics, USA). Assays were analyzed in triplicate. PCR was carried out at 95°C (30 s), followed by 95°C (5 s) and 60°C (20 s) for 40 cycles, and 95°C (15 s), 60°C (60 s) and 95°C (15 s). The 2^=ΔΔCT^ method was applied for data analysis. **Supplementary Table S1** shows the primers used.

### 2.13 Flow cytometry

Chondrocyte death was monitored with the caspase-1 fluorescence inhibitor probe FLICA 660-YVADFMK (BD Biosciences, USA). After chondrocyte digestion (EDTA-free 0.05% trypsin) and resuspension in 400 μL of binding buffer, the cells underwent double-staining with the membrane-linked protein V-FITC/propidium iodide (PI) staining kit (BD, Bioscience), which was utilized to detect pyroptosis (34). A FACSCalibur flow cytometer (BD Biosciences) was utilized for analysis with FlowJo (10.8.1). Experiments were performed in triplicate.

### 2.14 Western blot analysis

Chondrocytes in culture plates underwent lysis with the RIPA buffer (9806S, Cell Signaling Technology [CST]) containing 1 mM PMSF (ST506; Beyotime, China) and 1 mM phosphatase inhibitors (P1081; Beyotime). Each lysate was cleared by a 20-min centrifugation at 12,000 rpm/min and 4°C. Protein quantification was performed with a BCA Protein Assay Kit (Enhanced) (Beyotime). Equivalent quantities of total protein (30 μg) were resolved by 10% SDS-PAGE and subsequently wet-transferred onto a polyvinylidene difluoride (PVDF) membrane. Next, 5% bovine serum albumin (BSA) was employed for blocking (2 h) at ambient. After three washes with tris-buffered saline (TBS) containing 0.1% Tween-20 (TBST) for 10 min, overnight incubation was carried out with primary antibodies targeting Phospho-NF-κB p65 (1:1000; CST, #3033), NF-κB p65 (1:1000; CST, #8242), Phospho-IκBα (1:1000; CST, #2859), IκBα (1:1000; CST, #9242), iNOS (1:1000; Abcam, ab178945), COX2 (1:1000; Abcam, ab179800), Collagen II (1:1000; Abcam, ab34712), MMP13 (1:2000; Proteintech, 18165-1-AP), MMP3 (1:1000; Proteintech, 17873-1-AP), AhR (1:1000; Proteintech, 67785-1), ADAMTS-5 (1:1000; Abcam, ab41037), NLRP3 (1:1000; Proteintech, 19771-1-AP), Caspase-1/p20/p10 (1:4000; Proteintech, 22915-1-AP), GSDMD (1:1000; #AF4012, Affinity Biosciences), and GAPDH (1:20000; Proteintech, 10494-1-AP). Then, PVDF membranes underwent a 2-h incubation with goat anti-rabbit IgG H&L (HRP) (1:10,000; Abcam, ab6721) at ambient. An enhanced chemiluminescence kit (Millipore, USA) was employed for visualization. ImageJ v2.0.0-rc-69/1.52p was utilized to quantitate immunoreactive bands, relative to GAPDH levels.

### 2.15 Immunofluorescence

Chondrocytes seeded on coverslips in 24-well plates were grown to 70% confluence, pretreated with spermidine in the culture medium for 1 h, and incubated with or without IL-1β (10 ng/ml) for 24 h. After a 20-min fixation with 4% paraformaldehyde, permeabilization was carried out with 0.1% Triton X-100 (Solarbio Science & Technology, China) for 20 min, followed by a 30-min blocking of the treated chondrocytes with 10% normal goat serum (Servicebio, WGAR1009) at ambient. Following overnight incubation with antibodies against NF-κB p65 (1:500; CST, #8242) and collagen II (1:400; Abcam, ab34712) at 4°C, respectively, fluorescein-linked goat anti-rabbit IgG (1:500; Servicebio, GB21301) were added for 1h at ambient in the dark. The cytoskeleton was stained with YF^®^594-Phalloidin (1:150; Uelandy; China) for 15 min. DAPI counterstaining was performed for 5 min, and a confocal microscope (Olympus) was utilized for analysis.

### 2.16 Transmission electron microscopy

After adherent rat primary chondrocytes were gently scraped and fixed with 2.5% glutaraldehyde (PH7.4) for 2 to 4 h, mung bean-sized cell clusters were obtained after low-speed centrifugation (800 rpm, 5 min). Next, 1% osmium tetroxide was supplemented for gentle resuspension of the cell clusters. Ultrastructural analysis was performed with Hitachi 800 TEM (Japan) after dehydration, infiltration, embedding, and sectioning.

### 2.17 Histology

Knee joint samples were obtained from rats, fixed with 4% paraformaldehyde for one week, and transferred to a decalcification solution for six weeks, changing the solution every 2-3 days to remove calcium from the knee joints. Then, paraffin embedding and sagittal sectioning were performed. After cutting off 5-μm sections, staining was carried out with modified saffron-O and fast green, hematoxylin & eosin (H&E), or toluidine blue for histological examination. Histological assessment was carried out in a blinded fashion determining Osteoarthritis Research Society International OARSI scores (0-24) (35) and modified Mankin scoring system scores (0-14) (36).

### 2.18 Immunohistochemistry

Tissue sections underwent deparaffinization to water with xylene and graded alcohol, followed by brief washing with 0.1 M PBS (pH 7.4). Antigen repair was performed at 37°C for 30 min with an enzymatic antigen retrieval reagent (AR0026, Boster, Biological Technology, USA). Endogenous peroxidase activity was then quenched by incubating the samples with 3% H_2_O_2_ for 15 min. Anti-IL-6 primary antibody (1:200; Abmart, TD6087) was incubated at 4°C overnight. The remaining primary antibodies (1:200-500) were used as described above. Then, incubation was carried out with goat anti-rabbit HRP-linked secondary antibodies at 37ΔC for 1 h. The diaminobenzidine (DAB) substrate kit (8059, CST) was utilized for development for 3 min, followed by hematoxylin (G8550, Solarbio, China) counterstaining. The numbers and relative intensities of positive cells were assessed with Image J (National Institutes of Health, MD, USA).

### 2.19 Statistical analysis

Data are means ± standard error of the mean (SEM) and were analyzed using SPSS statistical software version 22.0 (SSPS, Inc., Chicago, IL). Results are expressed as means with 95% confidence intervals. Shapiro-Wilk’s and Levene’s tests were applied to assess normality and homogeneity of results, respectively. Statistical differences were determined using one-way analysis of variance (ANOVA) and analyzed using GraphPad Prism 9.4.1. *p*-values < 0.05 indicate statistical significance. To ensure the reliability of the findings, all experiments were biologically replicated at least three times.

## 3 Results

### 3.1 Molecular docking and dynamics

**Figure 1:**
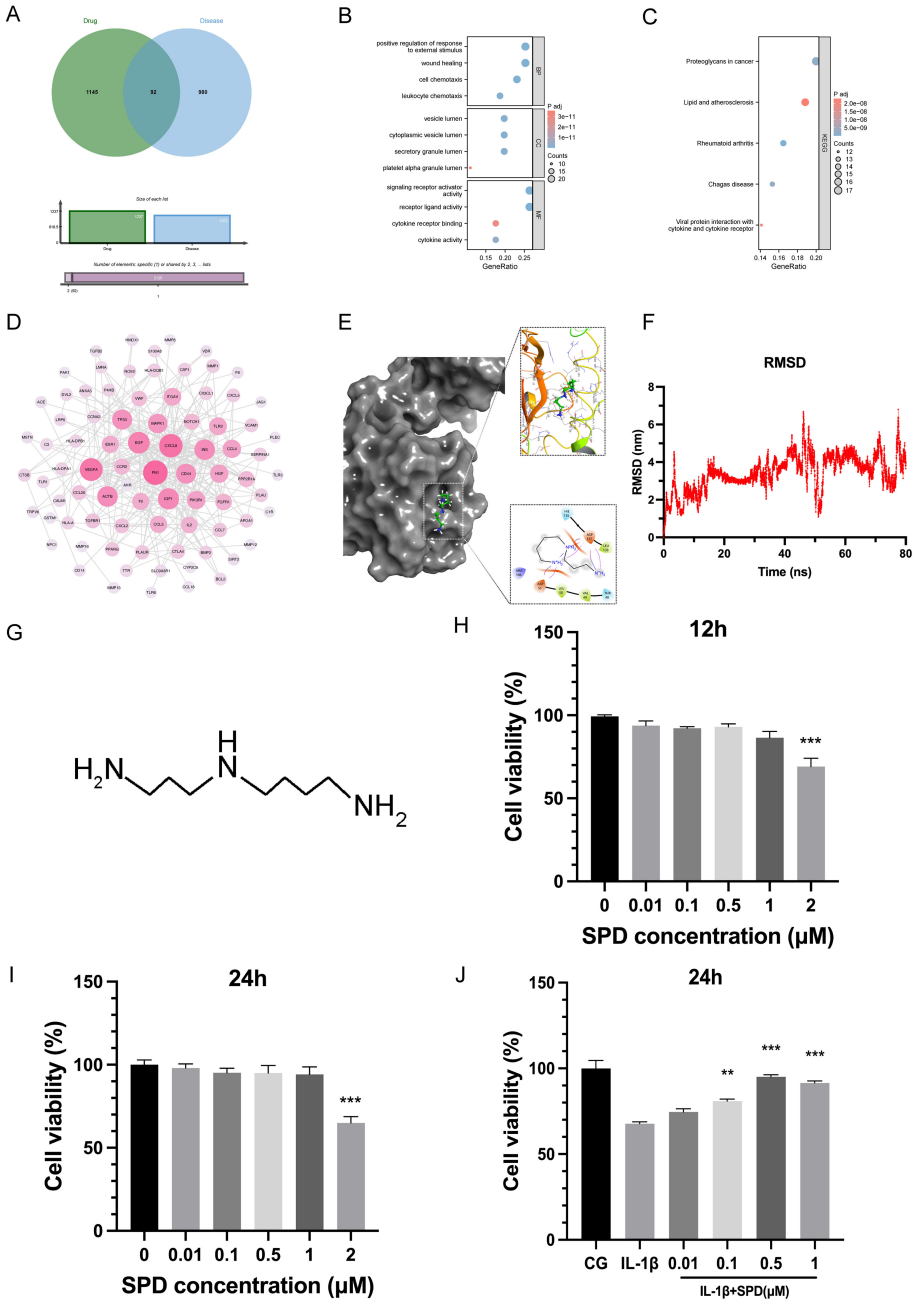
Effect of SPD on chondrocyte viability and molecular docking. **(A)** Venn diagram of potential targets of pharmaceutical interactions and information on osteoarthritis-related targets. **(B, C)** GO and KEGG enrichment analysis. **(D)** Protein-protein interaction (PPI) network analysis using the STRING database (https://string-db.org/). **(E)** Visualization of molecular docking results. **(F)** An 80-ns molecular dynamics (MD) simulation of the interaction between key targets and SPD. **(G)** Molecular structure of SPD. **(H-J)** Rat chondrocyte viability induced by 12h, 24h and IL-1β was assessed by CCK-8 assay using the indicated concentrations of SPD. *p < 0.05, **p < 0.01, ***p < 0.001.

By analyzing the OA-associated targets identified in the Genecards and Disgenet databases, we intersected the target prediction results of SPD with the potential therapeutic effects of SPD against specific targets (**Figure 1A**). These cross-targets provide significant insights into the mechanism underlying SPD’s treatment effect in osteoarthritis. In KEGG and GO enrichment analyses (**Figures 1B, C**), these targets were mainly involved in biological processes and pathways tightly associated with OA pathogenesis, and PPI network analysis (**Figure 1D**) further revealed the key positions and modes of action of these targets in the pathological network.

To examine SPD affinity for the target AhR, after visualization, a high-affinity hydrogen binding event was observed between SPD and AhR. The structure of AhR was obtained from the PubChem database, which provides a comprehensive structural database for protein analysis. In addition, space-filling modeling indicated that SPD was buried in the ligand-binding domains (**Figure 1E**). The results of molecular dynamics simulations showed that SPD formed stable interactions with the key target AhR and, as shown in **Figure 1F**, the RMSD values of SPD had low fluctuations during the simulations, indicating high structural stability. These findings suggest that SPD may affect the pathological process of OA by binding to AhR.

### 3.2 SPD’s effect on chondrocyte viability

**Figure 1G** shows SPD’s structure. Rat primary chondrocytes underwent pretreatment with SPD at 0, 0.01, 0.1, 0.5, 1, and 2μM for 12 h and 24 h, respectively. Subsequently, chondrocyte viability was assessed by the CCK-8 assay. **Figures 1H, I** revealed no significant cytotoxicity for SPD at 0-1μM. However, cell viability was reduced by IL-1β (10 ng/ml) treatment, and SPD could concentration-dependently reverse IL-1β-related cytotoxicity (**Figure 1J**). The most significant alleviating effect of SPD was observed at a concentration of 0.5μM, and therefore, 0.5μM SPD was selected for subsequent cell culture assays.

### 3.3 Anti-inflammatory capacity of SPD

**Figure 2:**
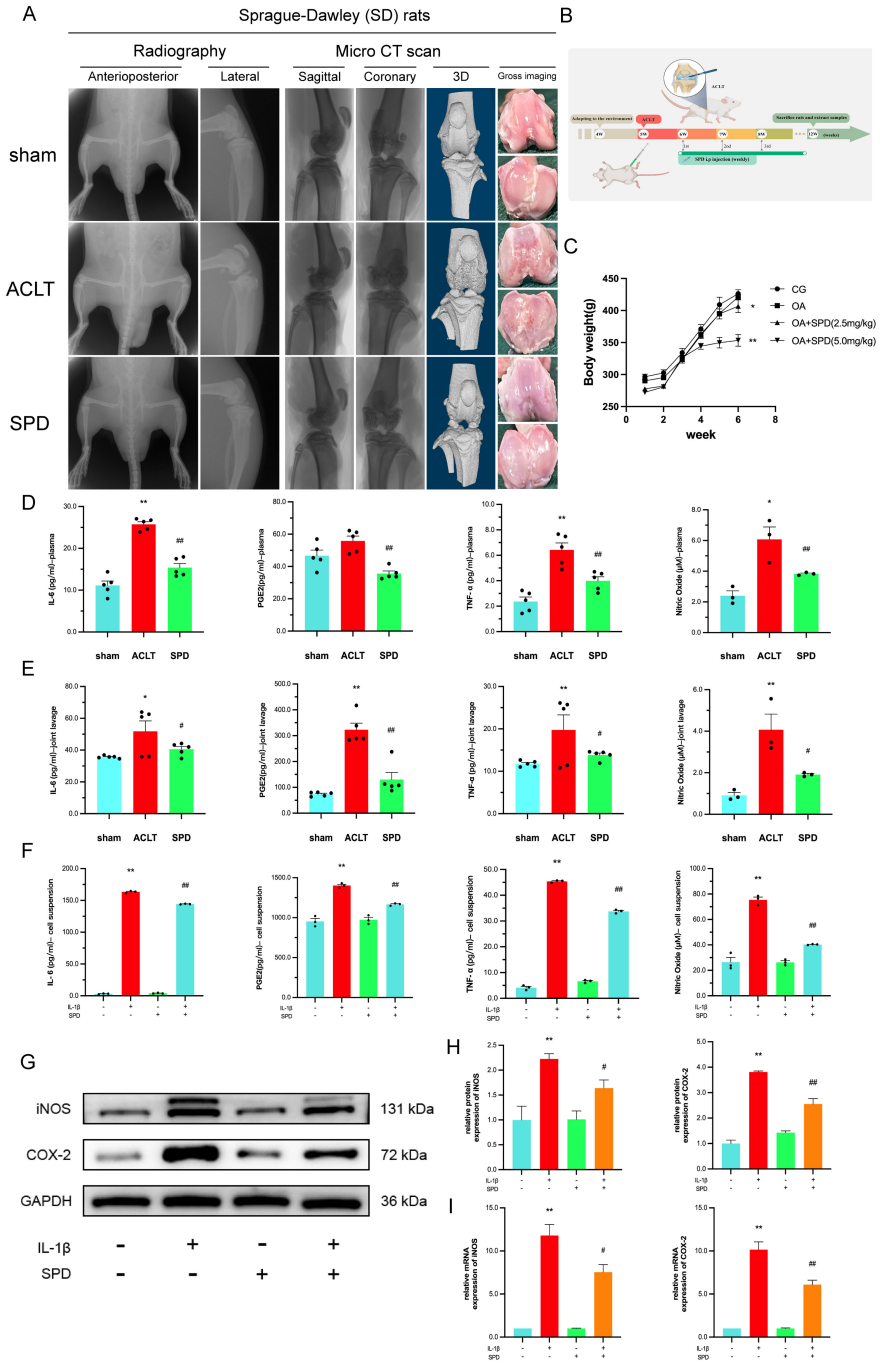
Inhibition of inflammatory factor production by SPD and imaging evaluation. **(A)** Gross imaging, plain radiography, and 3D CT reconstruction showed significant roughness of the articular surfaces, destruction of articular plane, and subchondral bone erosion in the ACLT group, whereas the SPD group had a more structurally intact joint, with less osteophyte generation and erosion. **(B)** Timeline and treatments for animal experiments. **(C)** Body weight changes in rats during the experimental period. **(D, E)** Inflammatory factors including TNF-α, IL-6, and PGE2 expression levels in rat serum and joint cavity lavage fluid were detected by ELISA, and NO content was measured by Griess reaction. **(F)** The levels of IL-6, TNF-α and PGE2 in cell suspensions were detected by ELISA, and the levels of NO were detected by Griess reaction. **(G, H)** Western blot and semi-quantitative analysis of iNOS and COX-2. **(I)** qRT-PCR analysis of iNOS and COX-2 in chondrocytes. *p < 0.05, **p < 0.01 vs. the control group; #*p* < 0.05, ##*p* < 0.01 vs. the IL-1β/ACLT group.

Following the adaptation of rats, a rat knee OA model was established by ACL transection at week 5. Each group received the corresponding treatment at 6 weeks of age. Radiographs, CT scans and 3D reconstruction images, as well as gross images of rats in each subgroup before sampling are shown in **Figure 2A**. The timeline and treatment methods of this study are shown in **Figure 2B**, and the rat weights recorded before each intervention are shown in **Figure 2C**. Our results showed that the ACLT group had significantly roughened articular surfaces, articular facet disruption, and subchondral bone erosions, while the SPD group had a more structurally intact joint, with less osteophyte and erosion. Studies have shown that daily administration of spermine to mice fed a high-fat diet (HFD) prevents obesity and improves glucose tolerance, and they found that exogenous administration of spermine resulted in a 24% reduction in body weight and an 18% reduction in fasting blood glucose (37). Our study showed that a dose of 5.0 mg/kg resulted in a more measurable reduction in body weight in rats. Next, based on the effect of SPD on body weight, we chose to evaluate the protective effect of a more effective concentration of SPD (5.0 mg/kg) on serum and arthritic lavage samples from rats. TNF-α, IL-6, and PGE2 amounts were quantitated by ELISA, while NO was detected by Griess reaction. TNF-α, IL-6, PGE2, and NO amounts were significantly elevated in plasma and joint cavity lavage fluid in the ACLT group in comparison with the sham group, whereas SPD reduced the amounts of inflammatory factors (**Figures 2D, E**, *p*< 0.05). To confirm SPD’s anti-inflammatory effect on chondrocytes, an IL-1β-induced cellular model was established. As shown in **Figure 2F**, IL-6, TNF-α, and PGE2 amounts were reduced in cell suspensions from the treatment group in comparison with the ACLT group (*p* < 0.05). Immunoblot and qRT-PCR showed SPD also reduced iNOS and COX-2 protein and mRNA amounts in IL-1β-induced chondrocytes (**Figures 2G-I**, *p* < 0.05). These findings suggested SPD could decrease the production of inflammatory cytokines.

### 3.4 Protective effect of SPD on the extracellular matrix in chondrocytes

**Figure 3:**
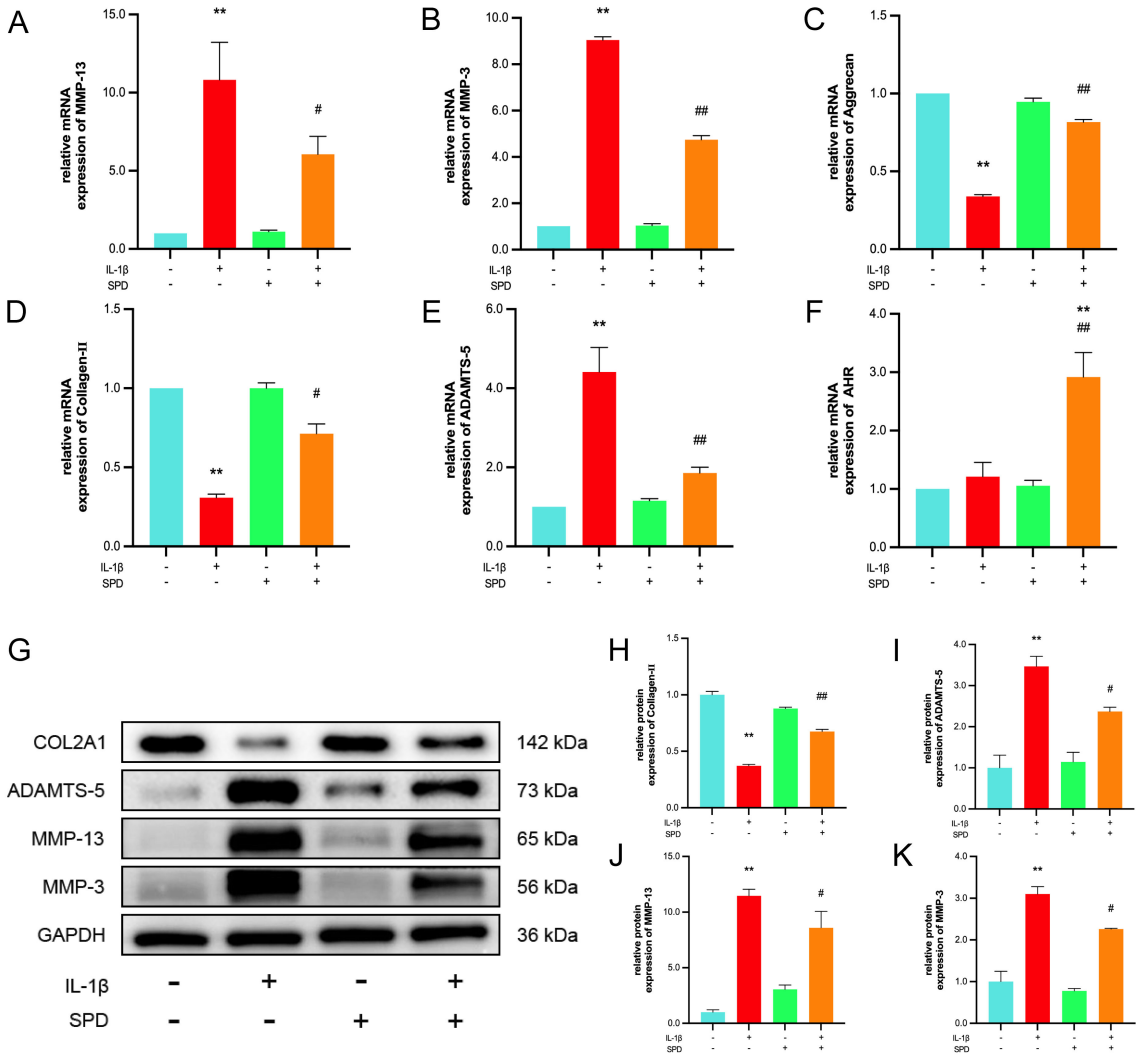
The protective effect of SPD on IL-1β-induced ECM degradation. **(A-F)** qRT-PCR analysis of MMP-3, MMP-13, ADAMTS-5, AhR, collagen-II and aggrecan. **(G-K)** Western blot and semi-quantitative analysis of COL2A1, MMP-3, MMP-13 and ADAMTS-5. *p < 0.05, **p < 0.01 vs. the control group; #*p* < 0.05, ##*p* < 0.01 vs. the IL-1β group.

Increased catabolism of articular cartilage ECM represents an important factor in OA development and progression (38). Pathological changes in cartilage ECM in osteoarthritis are dominated by the degradation of the functional matrix (most notably type II collagen and proteoglycans), occurring concomitantly with aberrant chondrocyte proliferation, senescence, inflammation, and hypertrophy (39). Therefore, SPD’s effects on the ECM in rat chondrocytes were examined. qRT-PCR revealed IL-1β elevated MMP-3, MMP-13, and ADAMTS-5 mRNA amounts and reduced type II collagen and aggrecan mRNA levels, whereas SPD inhibited these effects of IL-1β (**Figures 3A-E**, *p* < 0.05). Interestingly, as depicted in **Figure 3F**, addition of SPD upregulated the mRNA expression of AhR in response to IL-1β induction. Immunofluorescence of type II collagen, a characteristically expressed protein in rat primary cells, is shown in the **Supplementary Figure S1**. Immunoblot demonstrated SPD reversed the induction by IL-1β of catabolic factor-associated genes such as MMP-3, MMP-13, and ADAMTS-5, and elevated type II collagen amounts (40) (**Figures 3G-K**, *p* < 0.05). These findings suggested SPD reduced ECM degradation and promoted ECM synthesis.

### 3.5 Effects of SPD on NF-κB pathway effectors in osteoarthritis

**Figure 4:**
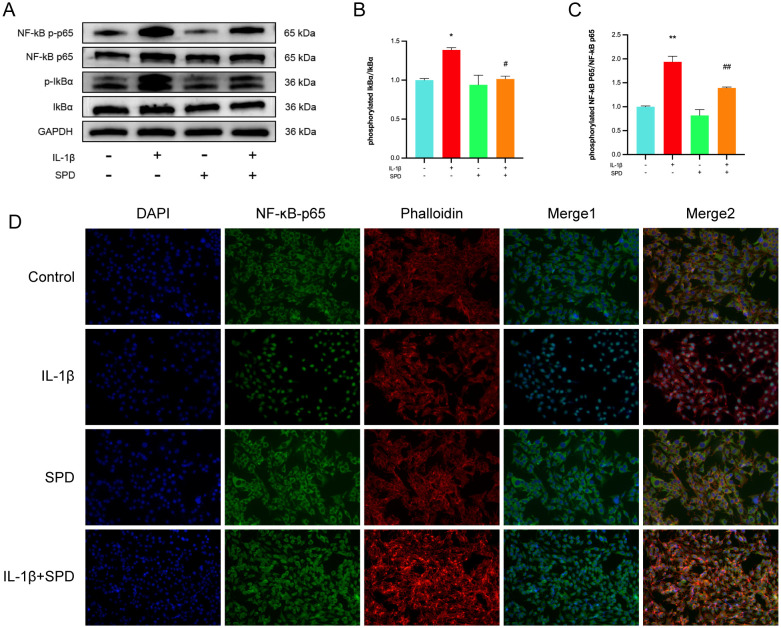
Effects of SPD on NF-κB signaling pathway. **(A-C)** Western blot and semi-quantitative analysis of p-IκBα, IκBα, p-p65, and p65. **(D)** Immunofluorescence to observe NF-κB P65 nuclear translocation. We stained the nuclei using DAPI (blue) and labeled NF-κB p65 using anti-NF-κB p65 rabbit fluorescent antibody (green), while labeling the cytoskeleton using YF^®^594-Phalloidin. Significant NF-κB p65 nuclear translocation was observed in the IL-1β group. pretreatment with SPD reversed IL-1β-induced nuclear translocation, whereas no significant changes were observed when SPD was applied alone. *p < 0.05, **p < 0.01 vs. the control group; #*p* < 0.05, ##*p* < 0.01 vs. the IL-1β group.

Multiple inflammatory genes are transcriptionally regulated by the nuclear factor κB (NF-κB) family of transcription factors, which are classically regulated by NF-κB induction via suppression of IκBs, resulting in the nuclear translocation of free NF-κB dimers. Immunoblot was carried out to detect NF-κB pathway effectors, measuring the expression of NF-κB p65 and Ikbα as well as their respective phosphorylated forms. Phosphorylated NF-κB p65 and Ikbα were markedly upregulated after IL-1β induction in comparison with the CG group; these changes were alleviated by the administration of SPD (**Figures 4A-C**, *p* < 0.05). Meanwhile, no significant changes in the phosphorylation levels were observed after addition of SPD alone (*p* > 0.05). To further explore NF-κB p65’s role in chondrocyte inflammation, immunofluorescence was performed to quantitate the nuclear translocation of NF-κB p65 (**Figure 4D**). NF-κB p65 translocation into the nucleus was enhanced after IL-1β treatment in comparison with the CG group, which was suppressed by SPD administration.

### 3.6 The anti-inflammatory role of SPD is AhR-dependent: regulation of NF-κB signaling

**Figure 5:**
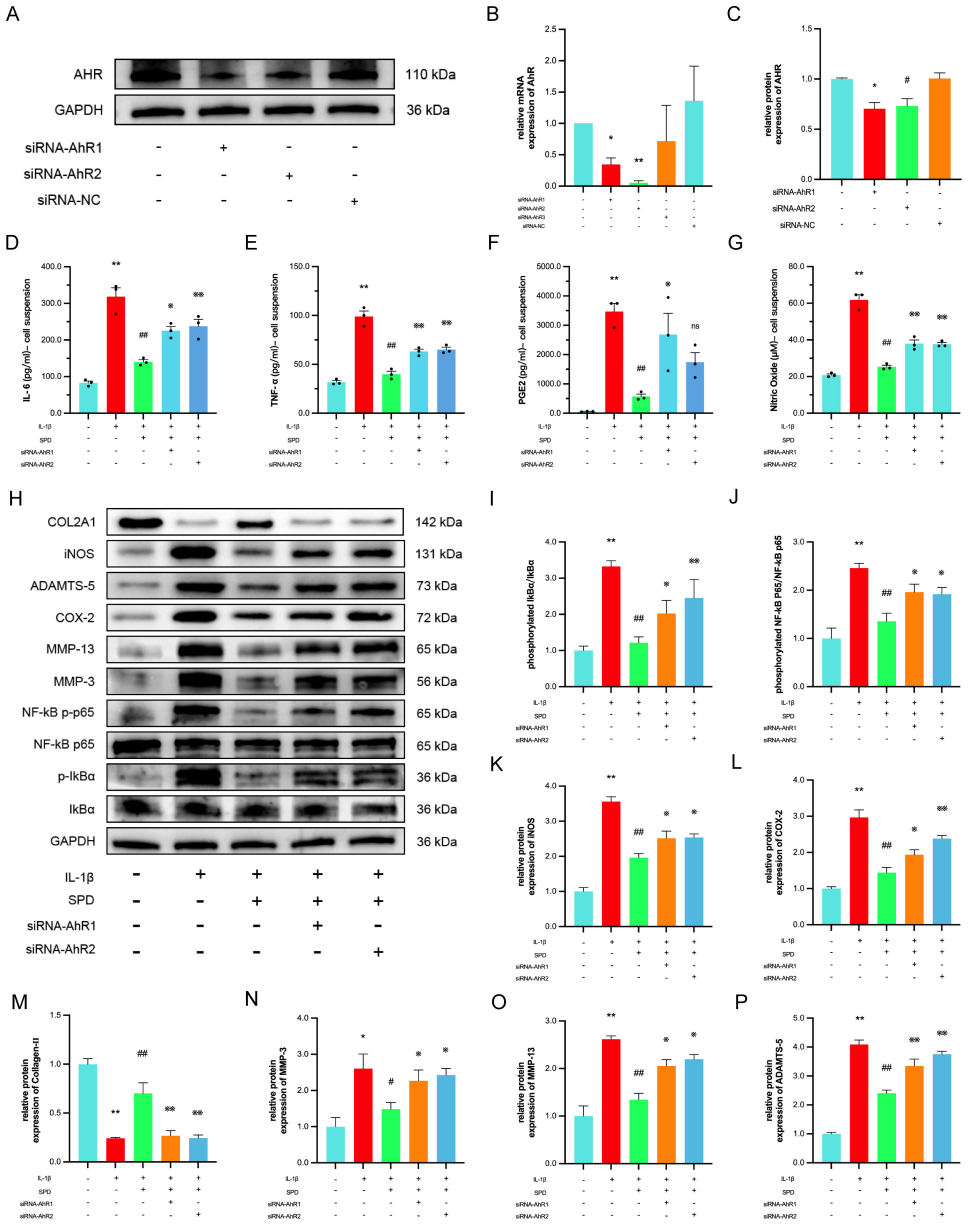
The protective effect of the SPD depends on the AhR. **(A-C)** Western blot and qRT-PCR analysis for AhR knockdown. **(D-G)** Expression of IL-6, TNF-α and PGE2 in cell suspensions by ELISA and NO by Griess reaction. **(H-P)** Western blot and semi-quantitative analysis of COX-2, iNOS, MMP-3, MMP-13, Collagen-II, ADAMTS-5 and p-IκBα, IκBα, p-p65 and p65. *p < 0.05, **p < 0.01 vs. the control group; #*p* < 0.05, ##*p* < 0.01 vs. the IL-1β group; ※*p* < 0.05, ※※*p* < 0.01 vs. the IL-1β+SPD group.

Spermidine exerts effective anti-inflammatory effects by increasing the intestinal mucosal barrier function via AhR in mice with experimental inflammatory bowel disease (IBD) (41). Combining our previous molecular docking data and molecular dynamics simulations, we suspect SPD likely exerts chondroprotective effects through AhR. Here, siRNA-AhR was employed to determine whether SPD exerts anti-inflammatory effects via AhR. To avoid the off-target effect of siRNA (42), two sequences with the highest knockdown efficiencies were selected from three siRNA sequences to knock down AhR. As depicted in **Figures 5A-C**, AhR mRNA and protein amounts were markedly reduced after chondrocyte transfection with siRNA-AhR (*p* < 0.05). ELISA and Griess reaction data for cell suspensions from each group after transfection revealed that SPD decreased TNF-α, IL-6, PGE2, and NO amounts, and such therapeutic effects were reversed by AhR knockdown (**Figures 5D-G**, *p* < 0.05). Western blot and qRT-PCR data (**Supplementary Figure S2**) demonstrated that SPD could reverse the changes induced by IL-1β on iNOS, COX-2, MMP-3, MMP-13, and ADAMTS-5, and upregulate collagen II and aggrecan (**Figure 5H**, *p* < 0.05). However, when AhR was knocked down, the alleviating effects of SPD on the above indicators disappeared (**Figures 5H-P**, *p* < 0.05).

Studies have shown that endogenous ligand-activated AhR suppresses NF-κB signaling periodontitis and IBD (43). Therefore, we investigated whether SPD affects the NF-κB inflammatory signaling pathway in chondrocytes by involving AhR. Immunoblot revealed SPD suppressed IL-1β-induced phosphorylation of IκBα and p65, whereas siRNA-AhR eliminated the inhibitory effects of SPD on NF-κB signaling (**Figures 5I-K**). The above data suggest SPD suppresses NF-κB signaling via AhR.

### 3.7 SPD exerts antipyroptotic effects by modulating NLRP3/caspase-1/GSDMD signaling

**Figure 6:**
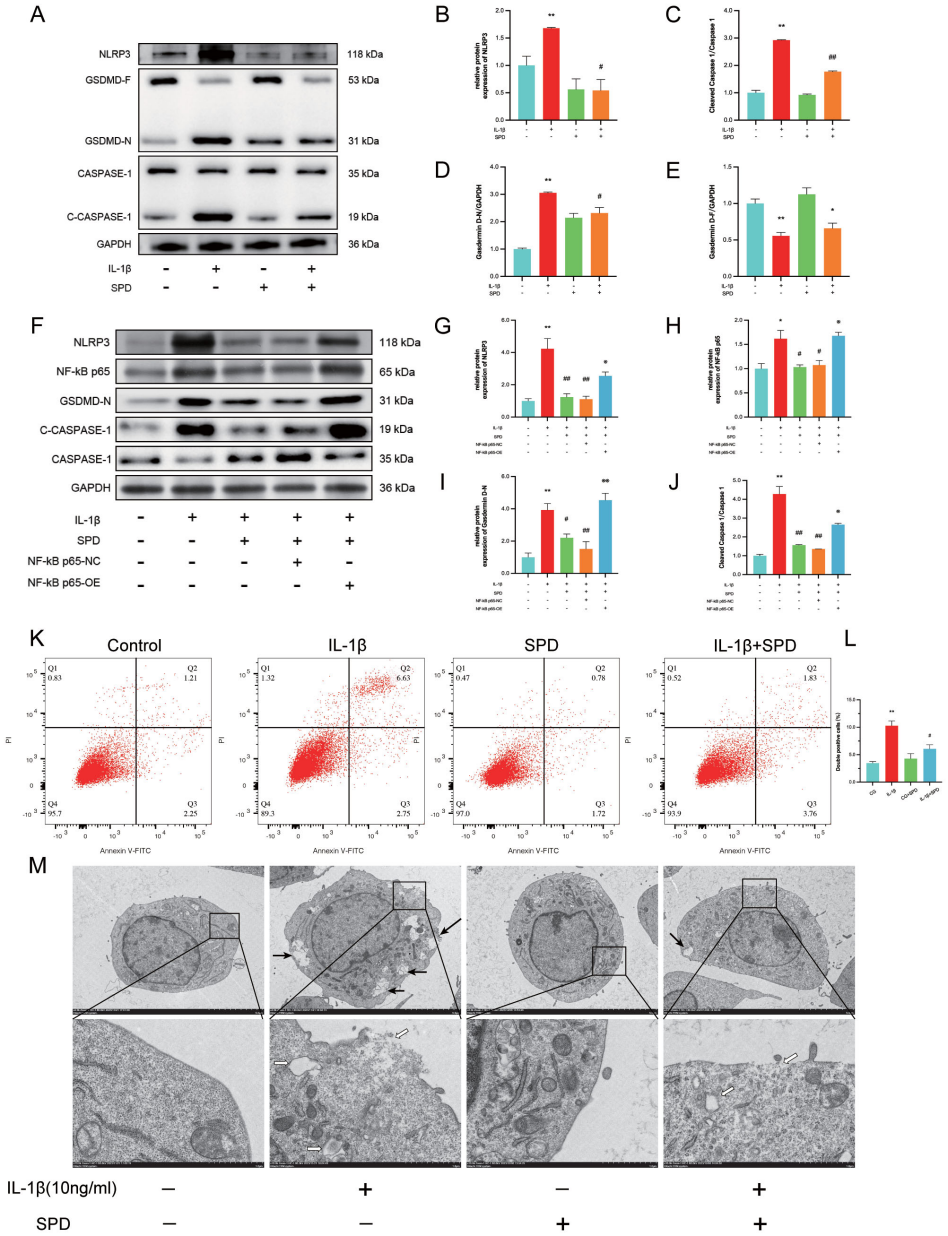
SPD ameliorates IL-1β-induced chondrocyte pyroptosis by inhibiting the NLRP3/caspase-1/GSDMD pathway. **(A-E)** Western blot measuring the differences in the expression levels of cellular pyroptosis-related proteins in each group. To elucidate whether SPD ameliorates chondrocyte pyroptosis by inhibiting AhR/NF-κB, NF-κB p65 overexpression plasmid was used to characterize the effect of the AhR/NF-κB pathway. **(F-J)** The inflammation-associated cellular pyroptosis proteins NLRP3, caspase-1, and GSDMD were significantly increased in the IL-1β group compared with the CG group, whereas downregulation of cellular pyroptosis was observed in the SPD group. Administration of P65 overexpression plasmid (OE group) blocked the therapeutic effect of SPD. **(K-L)** Flow cytometry showing chondrocytes labeled with caspase-1 fluorescence inhibitor probe FLICA 660-YVAD-FMK. IL-1β induces chondrocyte pyroptosis. SPD significantly alleviated IL-1β-induced chondrocyte death. **(M)** Transmission electron microscopy (TEM) images of chondrocytes from different treatment groups, such as cytoplasmic edema, swelling and rupture of cell membrane, nuclear consolidation, and organelle cavitation were more obvious in the IL-1β group; cell morphology of the SPD pretreatment group was significantly improved. Black arrows indicate cell membrane swelling; white arrows indicate organelle cavitation (Low magnification: ×2.0k, high magnification: ×10.0k). *p < 0.05, **p < 0.01 vs. the control group; #*p* < 0.05, ##*p* < 0.01 vs. the IL-1β group; ※*p* < 0.05, ※※*p* < 0.01 vs. the IL-1β+SPD group.

A recent study indicated that exogenous spermidine has a mitigating effect on inflammatory pyroptosis in diabetic cardiomyopathy in mice (44). We hypothesized that SPD plays an important role in the classical pathways involved in pyroptosis. Therefore, the mechanism by which SPD attenuates inflammation-associated pyroptosis in chondrocytes was examined. Immunoblot showed that SPD repressed NLRP3/caspase-1/GSDMD signaling effectors at the protein level (**Figures 6A-E**). IL-1β-induced NLRP3, gasdermin D-N terminus, and cleaved-caspase-1 were significantly upregulated compared with the CG group, and addition of SPD attenuated these effects.

To clarify whether SPD inhibits chondrocyte pyroptosis through the AhR/NF-κB pathway and thus inhibits chondrocyte pyroptosis, we observed changes in pyroptosis proteins using the NF-κB p65 overexpression plasmid. Western blotting results confirmed the NF-κB p65 overexpression plasmid effect (**Figures 6F, H**). Overexpression of p65 blocked the inhibitory effect of SPD on cellular pyroptosis proteins (**Figures 6F, G, I, J**).

To further assess the association of SPD with inflammatory pyroptosis, chondrocyte death was assessed by flow cytometry. SPD significantly (**Figures 6K, L**, *p*<0.05) alleviated chondrocyte pyroptosis. Transmission electron microscopy (TEM) provided a detailed examination of the cellular architecture, highlighting distinct features of pyroptosis. While the CG group displayed optimal cell morphology characterized by intact membranes and orderly organelles, the IL-1β-treated cells exhibited markers of pyroptosis, including cell membrane disruption and organelle disarray (**Figure 6M**). Notably, SPD treatment appeared to mitigate these changes, suggesting its role in preserving cell integrity under inflammatory conditions. Meanwhile, the IL-1β group tended to show cellular pyroptosis, with chondrocytes displaying cytoplasmic edema, cell membrane swelling and rupture, nuclear consolidation, cavitation of organelles, and thickening of the endoplasmic reticulum. However, SPD suppressed pyroptosis in SPD-treated chondrocytes, with improved chondrocyte morphology, reduced cell membrane swelling and organelle cavitation, as well as nuclear recovery. In conclusion, SPD was not only dependent on affecting the AhR/NF-κB signaling pathway, but also further inhibited the NLRP3/caspase-1/GSDMD signaling cascade, which alleviated IL-1β-related chondrocyte pyroptosis.

### 3.8 SPD attenuates OA progression in rats with experimental OA by downregulating inflammation-associated proteins

**Figure 7:**
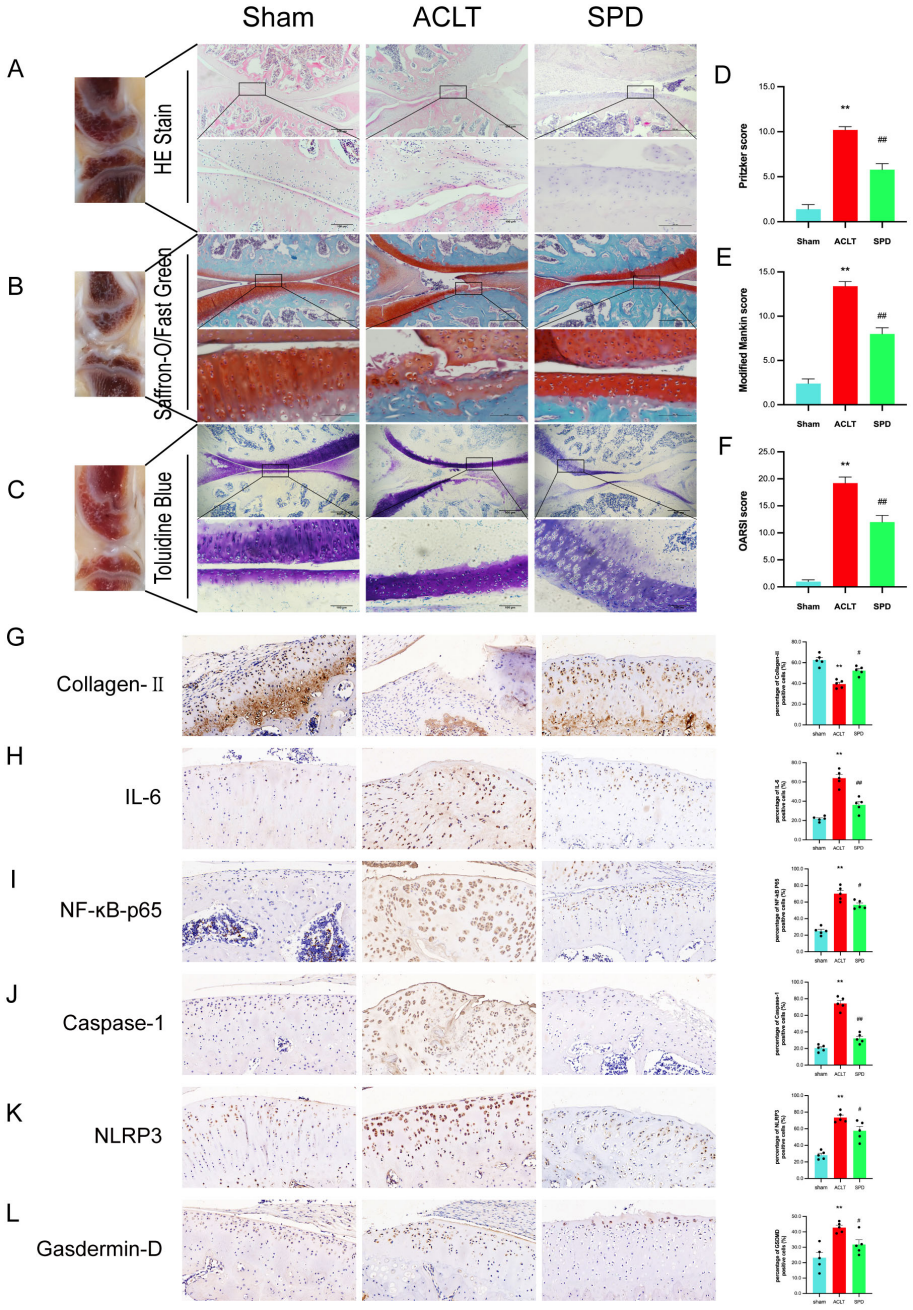
SPD attenuates the progression of OA in a rat model. **(A-C)** A representative image of H&E, saffron O-solid green and toluidine blue staining of cartilage sections from different groups. **(D)** Articular cartilage Pritzker score in different groups of rats. **(E)** The modified Mankin score for different groups of cartilage. **(F)** The cartilage OARSI scores of different groups. **(G-L)** Immunohistochemical staining and percentage of positive cells for IL-6, NF-κB p65, Caspase-1, NLRP3, Gasdermin-D and Collagen II. Scale bar = 20 μm, ×400. *p < 0.05, **p < 0.01 vs. the control group; #*p* < 0.05, ##*p* < 0.01 vs. the ACLT group.

To assess the potential protective role of spermidine in counteracting OA progression, SPD was administered via injection at weekly intervals in an ACLT rat model of OA. Histological analyses were conducted using hematoxylin and eosin (H&E), toluidine blue, and modified saffron-O/fast green staining to evaluate changes in cartilage and cellular structures (**Figures 7A-C**). Comparatively, the ACLT group exhibited notable cartilage degeneration, reduced chondrocyte density, and diminished matrix staining. In contrast, treatment with SPD resulted in a preservation of cartilage integrity and morphology, as evidenced by lessened cartilage destruction and a smoother cartilage surface, relative to the ACLT group. These observations are supported by improved Modified Mankin and Osteoarthritis Research Society International (OARSI) scores in the SPD-treated group (**Figures 7D-F**, *p*<0.01), indicating a mitigative effect on OA-related structural changes.

Immunohistochemistry results showed type II collagen amounts were markedly decreased in the ACLT group (*p* < 0.05), and these changes were reversed in the SPD group (**Figure 7G**). In addition, based on the percentage of positivity, IL-6 and NF-κB p65 levels were increased in the ACLT group versus sham animals, and these expression levels were significantly decreased after SPD injection (**Figures 7H, I**). In addition, among the inflammation-associated cellular pyroptosis protein markers (i.e., NLRP3, caspase-1, and GSDMD), an expression reversal of pyroptosis-related markers was detected in the SPD group (**Figures 7J-L**). Markers related to chondrocyte synthesis and catabolism are shown in **Supplementary Figure S3**. These data demonstrated that SPD was effective in attenuating OA progression and cellular pyroptosis *in vivo*.

## 4 Discussion

Various tissues rely on cells and the surrounding ECM for their function, which is important in OA development. In articular cartilage, for example, collagen II fibers and aggrecan act synergistically to resist compressive forces and impart elasticity (45). Type II collagen and aggrecan are essential for the maintenance of cellular phenotype in chondrocytes as characteristic markers (46). MMP-13, MMP-3, and ADAMTS-5 represent the major enzymes degrading the ECM, and elevated levels of matrix remodeling enzymes, such as matrix metalloproteinases (MMPs), induce the loss of normal phenotype in chondrocytes, which in turn indirectly controls joint remodeling (47). IL-1β upregulates iNOS and COX-2 in chondrocytes, increasing NO, PGE2, and TNF-α levels. Subsequently, PGE2 and NO upregulates MMP-3, MMP-13, and ADAMTS-5 (48). IL-1β also triggered the biosynthesis of further proinflammatory factors such as IL-6 and TNF-α, by induing NF-κB signaling, forming a positive feedback loop (49). This study found significant attenuation of chondrocyte inflammation and OA progression in a rat model after SPD administration. In addition, SPD significantly reversed IL-1β-induced upregulation of MMP-3, MMP-13, and ADAMTS-5 in chondrocytes, suggesting a potent suppressive effect of SPD on ECM degradation. These results were equally confirmed in the above gross image analysis, plain radiographs, 3D CT images, H&E micrographs, toluidine blue and Senna O staining, and immunobiological evaluation.

It has been shown that small molecule ligands can function by binding to the AhR receptor. Among them, AhR activation by endogenous ligands inhibits NF-κB signaling and reduces local inflammation in multiple conditions, including periodontitis, bronchitis, and colitis; Additionally, 5-HIAA activates AHR in B cells to promote regulatory B (Breg) cell differentiation and inhibit arthritis inflammation (50–52). To test our above hypothesis that SPD exerts its effect on OA progression through the AhR receptor, we first searched the database for information on OA targets and the drug’s molecular structure. Finally, 92 key targets were obtained through target prediction and screening. We analyzed the PPI network and found that AhR receptors may be involved in the process after SPD administration. Previous studies of drugs were mostly limited to static ligand-receptor molecular docking simulations (53), while this study incorporated dynamic simulations of drug action. Molecular dynamics simulation (MDS) has the advantage of providing more accurate and realistic information compared with *in silico* tools such as molecular docking, and MDS can overcome experimentally irrelevant results that may be induced by limited sampling of ligand and receptor conformations as well as approximate scoring functions (54). By simulating the movement of biomolecules on a time scale, MDS is not constrained by sampling limitations, providing more reliable data. In contrast, the above MDS data showed that spermidine formed a stable interaction with the key target AhR and maintained low fluctuations during the simulation, indicating high structural stability. Thus, we next explored the downstream pathway of SPD after its interaction with AhR.

NF-κB signaling is crucial for OA development, directly affecting cartilage matrix remodeling, chondrocyte apoptosis, and synovial inflammatory responses, while indirectly stimulating downstream regulators of chondrocyte terminal differentiation (55). This pathway is induced by the activation of pro-inflammatory factors, mechanical stress, and ECM degradation products. The NF-κB complex is phosphorylated and freely translocates into the nuclear compartment, triggering the transcription of downstream targets to regulate pro-inflammatory cytokines (56). As shown above, SPD starkly inhibited the phosphorylation of NF-κB pathway effectors and reduced the tendency of the NF-κB complex to undergo nuclear translocation in IL-1β-induced chondrocytes, and AhR silencing eliminated the above therapeutic effects of SPD, indicating SPD attenuates the inflammatory response via AhR/NF-κB signaling in IL-1β-treated chondrocytes.

**Figure 8:**
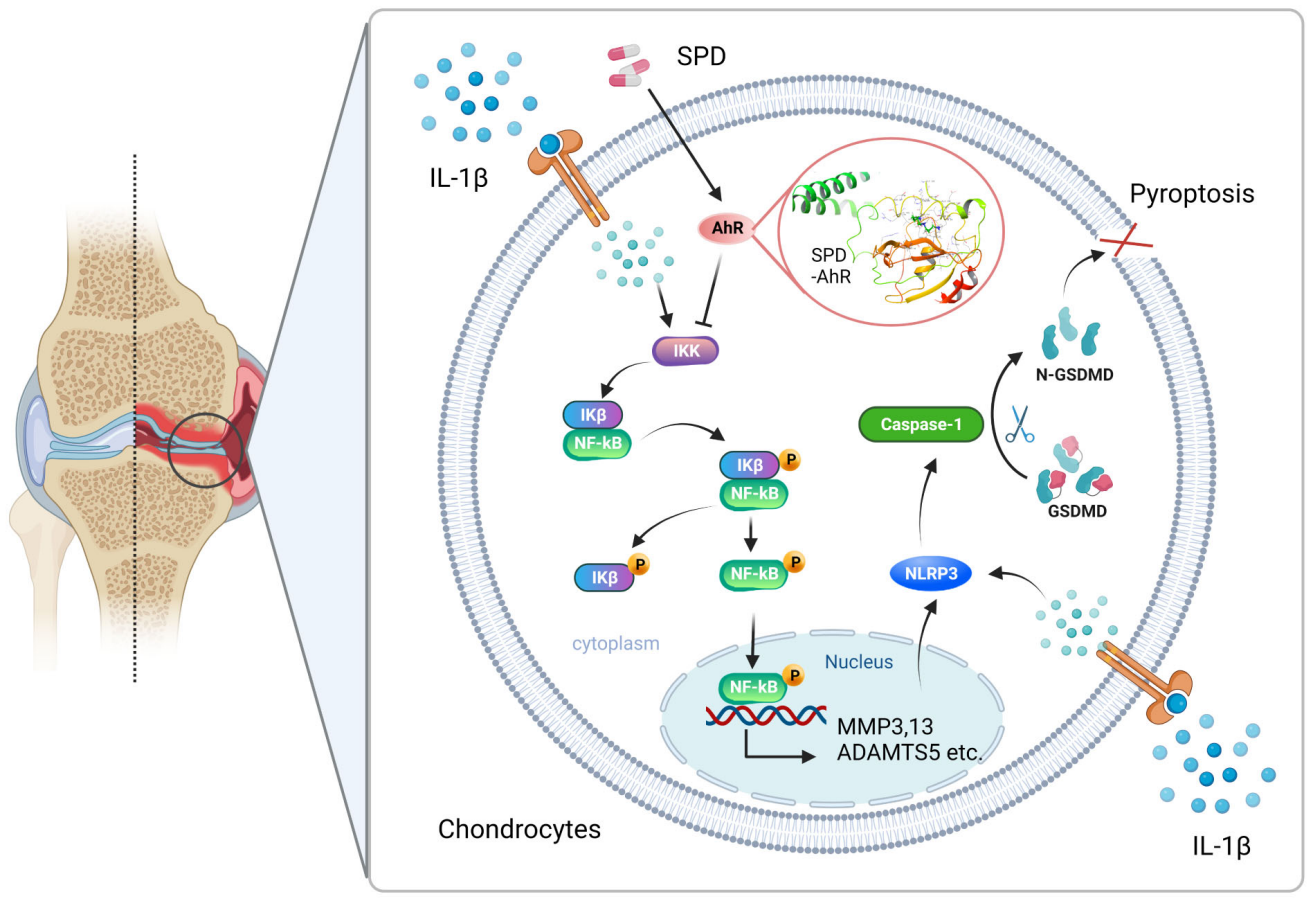
Mechanism of SPD inhibition of NF-κB signaling via AhR receptors and attenuation of chondrocyte inflammation and pyroptosis via NLRP3/caspase-1/GSDMD signaling pathway. A rat OA model was constructed using ACLT. Changes in the levels of inflammatory factors in animal samples were observed by intraperitoneal injection of SPD. We found that the drug entered the cells and bound to AhR targeting to inhibit chondrocyte inflammation and inflammation-associated cellular pyroptosis, thereby ameliorating the pathologic changes in OA. *In vitro*, SPD inhibited chondrocyte inflammation and inflammation-associated cellular pyroptosis by blocking the activation of AhR/NF-κB and NLRP3/caspase-1/GSDMD signaling pathways in IL-1β-treated chondrocytes.

In Ma et al.’s study, *in vitro* experiments used IL-1β to induce the formation of cellular pyroptosis and the upregulation of NLRP3 inflammasome (57). A positive feedback exists between IL-1β, a proinflammatory factor, and the cascade response of the NLRP3 inflammasome. Chen and colleagues (58) first found that IL-1β could promote its own production by enhancing the activation of the NLRP3 inflammasome. Pyroptosis is activated via the classical NLRP3/caspase-1/GSDMD cascade, which is involved in OA’s pathological process (59). Among them, caspase-1-associated GSDMD cleavage enhances membrane perforation and cell cleavage, which provides clues to the necrotic execution mechanism of cellular pyroptosis (60). Wang et al. synthesized that IL-1β induces inflammatory responses, stimulates ECM degradation, accelerates cellular senescence, and induces apoptosis and pyroptosis in IVD cells, whereas the disc and the cartilage are both aneural and avascular tissue in body (61). Similarly, previous studies by our team using 10 ng/mL of IL-1β induced primary chondrocytes in rats revealed that irisin and the novel adipocytokine metrnl inhibited the activation of the nodule-like receptor protein-3/caspase-1/gasdermin D cascade, improving the process of chondrocyte pyroptosis (33, 62). Our study confirms the potential mechanism underlying the anti-pyroptosis effect of SPD in an osteoarthritis cell model, revealing for the first time the potential of SPD to alleviate pyroptosis. Notably, SPD intervention alleviated OA by inhibiting inflammation and pyroptosis mediated by the AhR/NF-κB and NLRP3/caspase-1/GSDMD cascades. Our proposed mechanism is elucidated in detail in **Figure 8**.

In the present work, SPD significantly suppressed IL-1β-induced inflammation, attenuated ECM degradation, and upregulated matrix synthesis proteins. SPD administration was accompanied by NF-κB signaling inactivation. When AhR was knocked down in chondrocytes, the protective ability of SPD was blunted, demonstrating SPD’s anti-inflammatory ability depends on AhR. In addition, the anti-pyroptotic effect of SPD was achieved by inhibiting the NLRP3/caspase-1/GSDMD cascade. Furthermore, SPD effectively attenuated OA progression in a rat model. These results are consistent with several recent studies, however, interestingly, other studies have also raised doubts in the pathway of effects of SPD on OA. Stefania D’ Adamo et al. reported that SPD can exhibit anti-oxidative stress and anti-inflammatory protective effects on cartilage by rescuing autophagic fluxe (32), however, a more recent study by Ou et al. found that SPD has no direct protective effect on chondrocytes *in vitro* but could ameliorate articular cartilage degeneration by modulating M1/M2 polarization of synovial macrophages (63). Also polyamine metabolism regulates a novel form of cell death, ferroptosis, mediated through P53 (64). This discrepancy highlights the complexity of OA pathogenesis and suggests that the possible role of SPD in the intra-articular microenvironment deserves further investigation.

Although our study revealed the potential mechanisms by which SPD inhibits chondrocyte inflammation and cellular pyroptosis *in vitro* and *in vivo* via the AhR/NF-κB axis and the NLRP3/caspase-1/GSDMD pathway, its clinical application still faces several limitations. First, although this study was based on the rat ACLT model, which is commonly used in osteoarthritis research, the model does not fully mimic the pathological process of human OA. Human OA is usually the result of a combination of age, genetics, mechanical load, and intra-articular environment (including chondrocytes, synoviocytes, subchondral bone, and infrapatellar fat pads, etc.), whereas the ACLT model mainly mimics traumatic osteoarthritis, and therefore whether the results of the present study can achieve similar therapeutic effects in patients with multifactorial OA needs to be verified by other animal models and clinical trials. Secondly, this study used *in vivo* SPD treatment in rats, while the pharmacokinetics and different modes of administration (intra-articular injection) in humans as well as the optimal dose and therapeutic window of SPD in humans are the focus of future studies. In conclusion, these future studies will help to clarify the prospects of SPD in OA patients and provide a theoretical basis for the development of novel OA treatment strategies.

## 5 Conclusion

This report provides new insights into the potential functional dietary therapeutic use of the natural polyamine SPD in the treatment of OA, while highlighting the anti-inflammatory capacity of ligand-activated AhR, providing new perspectives for high-throughput screening of small-molecule compounds for the treatment of OA.

## Data Availability

The original contributions presented in the study are included in the article/**Supplementary Material**. Further inquiries can be directed to the corresponding author.
