# Supplementary material for: Spermidine attenuates chondrocyte inflammation and cellular pyroptosis through the AhR/NF-κB axis and the NLRP3/caspase-1/GSDMD pathway
Source: Front Immunol. 2024 Oct 2;15:1462777. doi: 10.3389/fimmu.2024.1462777 (PMC11479918; doi:10.3389/fimmu.2024.1462777)
Supplement: Supplementary file 1 [file DataSheet1.docx]

**Table S1** Sequences of primers used for real-time PCR.

| **Gene symbol** | **Sequence (5´- 3´)** |
| --- | --- |
| MMP13-F | AAGGTCTGGTCTGATGTGACAC |
| MMP13-R | AAAAGCGTGTGCCAGAAGAC |
| COL2A1-F | ACGCCATGAAAGTCTTCTGC |
| COL2A1-R | CAGATGTGCTTCTTCTCCTTGC |
| Aggrecan-F | AGGGTCAGGAGAAACAGAGTCAACC |
| Aggrecan-R | AGCATGTGAAAGTGTCCAAGGCATC |
| AHR-F | CCTAAGCAAGTTTCAGAGTCCGAGC |
| AHR-R | CTACAGGAATCCGCTGGGTGTGATA |
| COX-2-F | GAAACCTAGCACCTTCGGAGGAGAA |
| COX-2-R | GCACATTGAAAGAGGCAAAGGGACA |
| iNOS-F | CTACCACTTTGAAGAAGCTGGTGGC |
| iNOS-R | TTCTGGCTCTTGAGCTGGAAGAAGT |
| ADAMTS-5-F | GTGGAGTGTGTGGAGGGGATAACTC |
| ADAMTS-5-R | TTATGTGGGTTGCTCCTTCAGGGAT |
| MMP-3-F | AGAACTTTCCAGGCATTGGCACAAA |
| MMP-3-R | TCCAACTGTGAAGATCCGCTGAAGA |
| GAPDH-F | ACAACTTTGTGAAGCTCATTTCCTGGT |
| GAPDH-R | GGCCTCTCTCTTGCTCTCAGTATCC |

**Supplementary materials Figure 1**

Microscopy and immunofluorescence analyses of type II collagen were employed to identify primary chondrocytes. The cell morphology of each subgroup was observed under the microscope as shown in Figure 3A, in which we performed immunofluorescence identification of type II collagen with the untreated Control cells group (Figure 3B) to demonstrate the effectiveness of primary cell extraction.

**(A)** Observation of the morphology of primary chondrocytes in each subgroup by inverted phase contrast microscopy (Low magnification: ×100, high magnification: ×200).

**(B)** Immunofluorescence and identification analysis of Collagen II (scale bar = 100 μm, ×200).


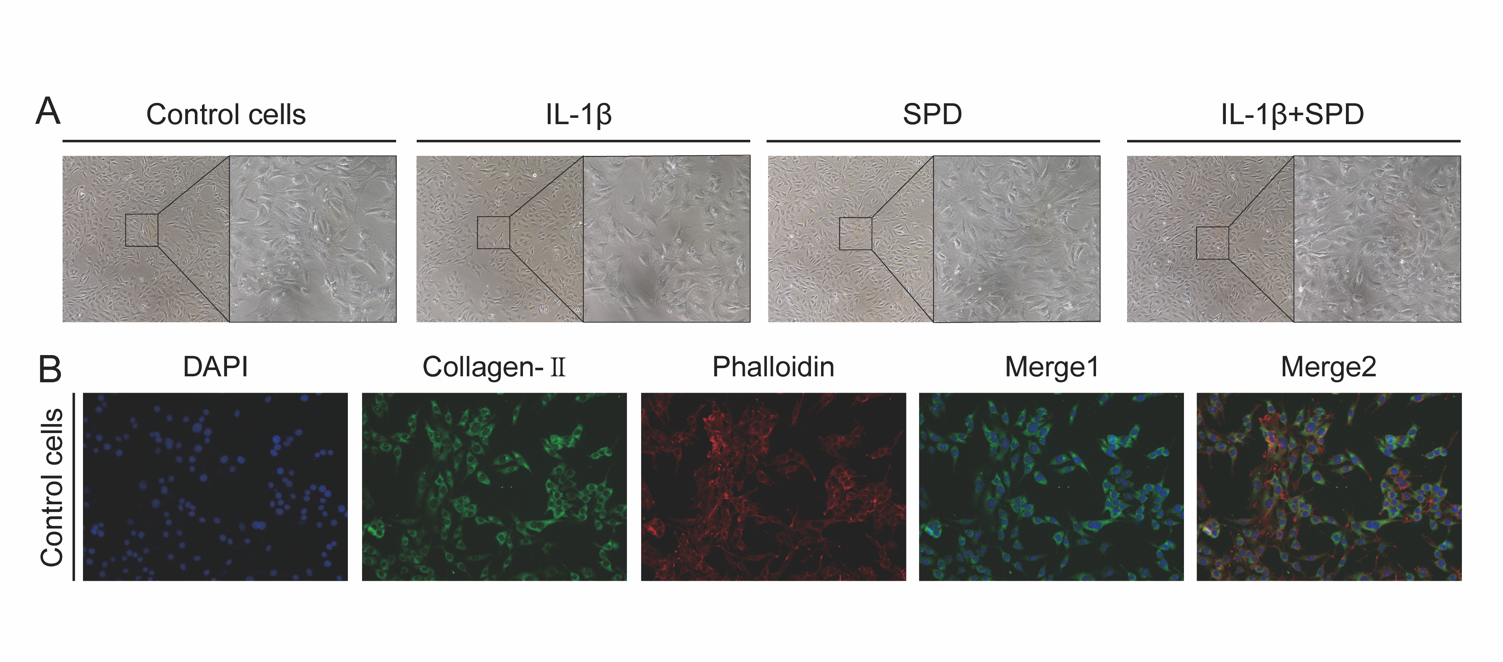


Supplementary materials Figure 1

**Supplementary materials Figure 2**

**(A)** qRT-PCR analysis of iNOS, COX-2, MMP-3, MMP-13, ADAMTS-5, Collagen-II, and Aggrecan.

**
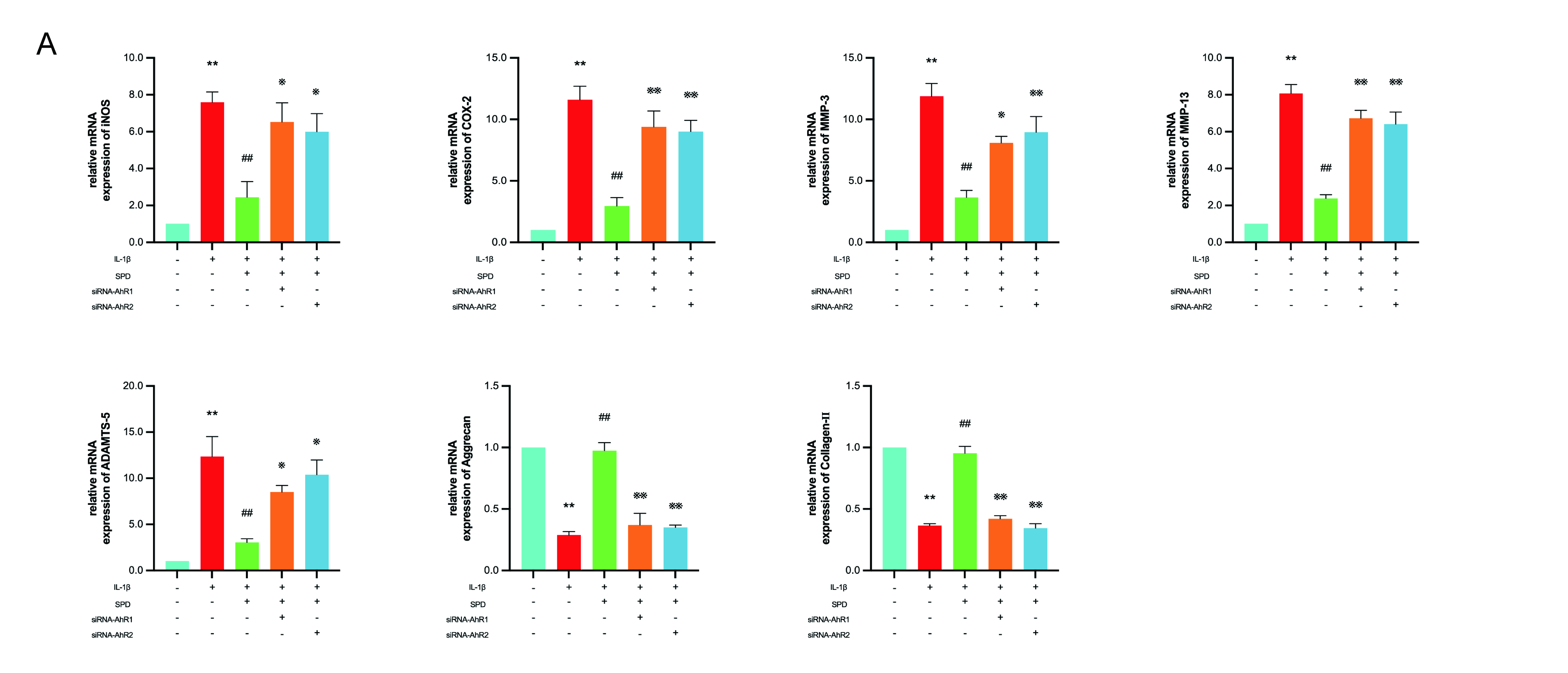
**

Supplementary materials Figure 2

**Supplementary materials Figure 3**

**(A-D)**: Markers of cartilage-related synthesis and catabolism in osteoarthritis, including Aggrecan, ADAMTS-5, MMP3, MMP13.

**
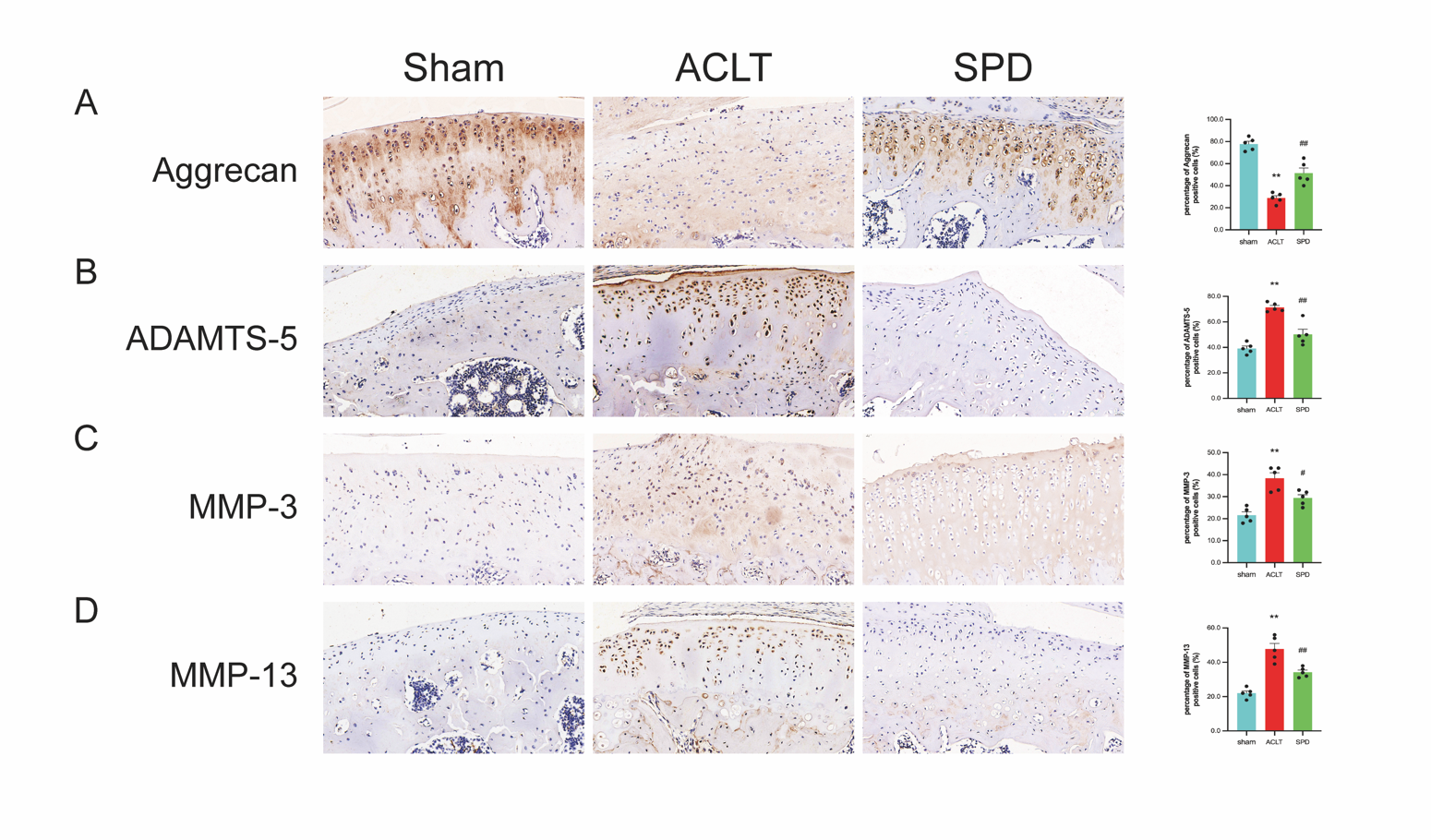
**

Supplementary materials Figure 3
